# PDX: Moving Beyond Drug Screening to Versatile Models for Research Discovery

**DOI:** 10.1210/jendso/bvaa132

**Published:** 2020-09-12

**Authors:** Gail P Risbridger, Mitchell G Lawrence, Renea A Taylor

**Affiliations:** 1 Department of Anatomy and Developmental Biology, Biomedicine Discovery Institute Cancer Program, Monash University, Melbourne, Victoria, Australia; 2 Prostate Cancer Research Program, Cancer Research Division, Peter MacCallum Cancer Centre, Melbourne, Victoria, Australia; 3 Sir Peter MacCallum Department of Oncology, University of Melbourne, Melbourne, Victoria, Australia; 4 Department of Physiology, Biomedicine Discovery Institute Cancer Program, Monash University, Melbourne, Victoria, Australia

**Keywords:** prostate cancer, breast cancer, patient-derived xenograft, organoid, pathology, preclinical testing

## Abstract

Patient-derived xenografts (PDXs) are tools of the trade for many researchers from all disciplines and medical specialties. Most endocrinologists, and especially those working in oncology, commonly use PDXs for preclinical drug testing and development, and over the last decade large collections of PDXs have emerged across all tumor streams. In this review, we examine how the field has evolved to include PDXs as versatile resources for research discoveries, providing evidence for guidelines and changes in clinical practice.

## 1. Materials and Methods

Articles included in this narrative review were compiled from original research articles, society guidelines, and reviews from peer-reviewed journals included in the PubMed database as of July 15, 2020. Search terms included “prostate cancer” OR “breast cancer” AND “patient-derived xenograft” OR “preclinical model” OR “patient-derived model” AND either “organoid,” “explant,” OR “slice culture.” We additionally searched references within publications relevant to the topic.

## 2. What is a Patient-Derived Xenograft (PDX)? One-Time vs Serially Transplantable PDXs

For decades, cell lines have been essential elements of laboratory research. Many of us are familiar with the NCI-60 cancer cell line panel that was established to provide a diverse and thoroughly characterized set of cell lines for screening therapeutic compounds [1]. Without doubt, the panel enabled successful drug development. But in 2020, the term *diversity* includes the notion that it is imperative to match a drug to a patient, or group of patients, most likely to respond; that is, the need for precision medicine. This is where the use of patient-derived models, such as xenografts, has emerged.

**Figure 1.**
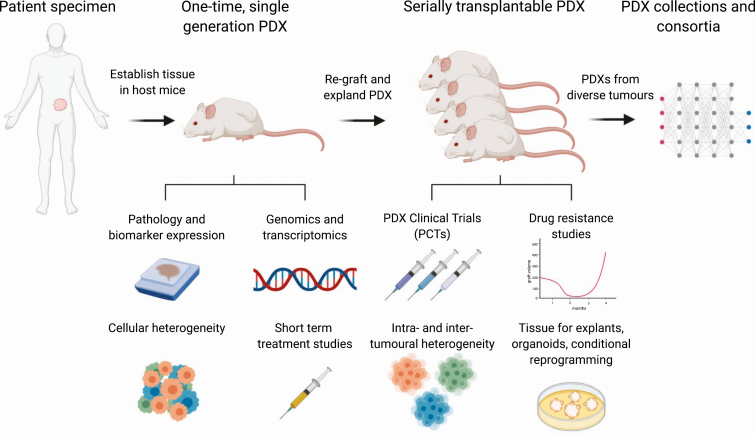
Utility of patient-derived xenografts for discovery research of hormone-dependent cancers. Collaborations between clinicians and scientists provide access to the high-quality human tumor specimens that are critical for establishing patient-derived xenografts (PDXs). Tumors can be grown as first-generation or one-time PDXs, which are useful for studying the short-term expansion of human tumors; pathology and biomarker expression; the genomic and transcriptomic features of tumors from rare patient cohorts; cellular heterogeneity; and the response of tissues to hormone manipulation and other short-term treatments. Serially transplantable PDXs are useful for drug testing in PDX clinical trials (PCT); identifying mechanism of therapy-resistance that evolve over time; studying intertumor and intratumor heterogeneity; and providing renewable sources of material for explants, organoids, and cultures of conditionally reprogrammed cells. Large collections of serially transplantable PDXs with diverse phenotypes are stored and disseminated by consortia. Altogether, these different uses of PDXs increase the capability and impact of preclinical and translational studies, providing evidence for changing guidelines and improving the clinical management of hormone-dependent cancers.

A patient-derived xenograft (PDX) is a specimen taken from a patient and engrafted into a host mouse so that the tumor tissue remains viable for weeks or months, often surpassing its longevity in 2D in vitro culture [2]. The specimen may be a biopsy, a piece of tissue from a primary tumor or metastasis, ascites, effusions, or circulating tumor cells [3-7]. The sample is grafted into host mice at an orthotopic or heterotopic site (into the muscle or bone, or under the skin or renal capsule) [8-11]. The host mice are usually immunosuppressed to reduce tissue rejection. The resultant PDX may be used one time, and studied while it survives in the original graft site as a single-generation PDX, or it may be serially transplanted, by harvesting the first graft and regrafting a portion to regrow in subsequent host mice (Fig. 1). Repeating the process produces generations of a PDX, and many serially transplantable PDXs can survive for years in host mice. Cryopreserving serially transplantable PDXs can extend their longevity even further [12]. As new serially transplantable PDXs are established, they become useful models for preclinical testing and discovery research. Indeed, in response to the continuing need for more diverse models, the National Cancer Institute established a national repository of patient-derived models, including PDXs, cell cultures, organoids, and cancer-associated fibroblasts [13].

## 3. Serially Transplantable Patient-Derived Xenografts as Preclinical Models of Hormone-Dependent Cancers

Serially transplantable PDXs enable researchers in academia and industry to share resources and reduce duplication of effort. Studies using serially transplantable PDXs for preclinical testing are elaborate, time consuming, and expensive, but they may facilitate the translation of promising agents to clinical trials. It is hoped that conducting PDX clinical trials in host mice will inform the design of clinical trials, accelerate translation, and reduce the failure rate of new oncology drugs [14]. Thus, over recent years, we have come to expect that data from PDXs will be provided as preclinical evidence of drug efficacy.

Large consortia that span tumor streams, such as EurOPDX, Novartis, The Jackson Laboratory (JAX), CrownBio, and the National Cancer Institute’s PDXNET and Patient-Derived Models Repository, are able to identify therapeutic targets and biomarkers for specific diseases or different malignancies [13-15]. Smaller consortia, such as those for breast and prostate cancers, are more specialized and reflect the need to understand the evolution of endocrine therapy-resistant tumors as new hormonal therapies come into practice.

In 2016, a consortium of academic laboratories from Europe, Australia, and North America reported a collection of 537 serially transplantable PDXs, representing all 3 clinical subtypes of breast cancer: estrogen receptor positive (ER+), HER2+, and triple-negative breast cancer (TNBC) [16]. In this collection, 56% of PDXs were from TNBC, 36% from ER+ cancer, and only 8% from HER2+ breast cancer. ER+ tumors are the most commonly diagnosed subtype and readily accessible specimens, but they are frequently low grade, more differentiated, and have lower Ki67, leading to low engraftment rates. In comparison, the more aggressive TNBC tumors clearly have a higher take rate as PDXs [16].

For prostate cancer, the largest collection of PDXs was reported by the Movember GAP1 PDX consortium, with 98 serially transplantable PDXs [17]. This included PDXs of castration-sensitive and castration-resistant primary and metastatic prostate adenocarcinomas, as well as tumors with neuroendocrine differentiation. The prostate cancer PDXs within this collection were established by individual research laboratories, and the investigators formed the international consortium with the goal of providing the PDXs to the prostate cancer research community [17].

In breast and prostate cancer, the most aggressive and difficult to treat tumors are ER negative (ie, TNBC) and androgen receptor (AR) null (ie, neuroendocrine prostate cancer). These tumors do not respond to hormone-directed therapies, so new targeted therapies are urgently required. Thus, there is a significant need to establish serially transplantable PDXs of hormone-independent breast and prostate cancers to make these important discoveries.

## 4. The Utility of One-Time, Single-Generation Patient-Derived Xenografts as Tools for Research Discovery

Beyond preclinical testing, an important use of PDXs for research discovery that is often overlooked is engrafting them for one-time only use. These single-generation PDXs can be integrated with clinical research, and lead to rapid changes in clinical practice. This is particularly relevant to the prostate cancer field, where it is challenging to establish serially transplantable PDXs.

One advantage of using single-generation PDXs is that most tumors initially survive in host mice, with reported take rates of first-generation grafts in the range of approximately 40% to 90% [9, 18-20]. Although there are no standard criteria for assessing the take rate, it is often based on the presence of proliferating cancer cells in grafts after they are harvested. Because tumor samples survive as single-generation PDXs for at least 6 to 8 weeks, this approach provides an opportunity to test short-term treatments and examine changes in tumor growth. Long-term propagation remains the major challenge because proliferation is often very low in prostate cancer grafts. Prostate tumors often lack the rapid proliferation and exponential tissue expansion required to generate serially transplantable PDXs, so less than 20% of samples produce long-term PDXs [21].

Another feature of single-generation PDXs is that they maintain the complex composition of the original tissue, including benign cells and different growth patterns of cancer. Therefore, single-generation PDXs can yield important insight about the original specimen. For example, in collaboration with the Kathleen Cuningham Foundation Consortium for Research into Familial Aspects of Breast Cancer (kConFab), we obtained radical prostatectomy specimens from men with germline mutations in *BRCA2* and grew them as one-time PDXs [22]. We identified intraductal carcinoma of the prostate as a prevalent pathology in these PDXs, sparking further investigations that showed the frequency of this pathology was as high as 42% in men with germline *BRCA2* mutations [22]. Further, we demonstrated that intraductal carcinoma of the prostate was associated with significantly poorer outcomes in men with germline *BRCA2* mutations [22]. This research discovery was made in a rare cohort of patients with germline mutations, and although the outcome did not directly affect pharmacological development, significant changes in clinical practice and management of patients with these clinical features were included in oncology guidelines [23].

Another application of one-time PDXs was the study of castrate-tolerant cells. Androgen deprivation therapy is the mainstay treatment for advanced prostate cancer, but it is postulated that some cancer cells are inherently resistant to this therapy because castrate-resistant prostate cancer eventually emerges. Thus, tumor cells that survive androgen withdrawal are critical cellular targets for more effective treatments in prostate cancer. Using prostate cancer specimens from unselected men, we successfully grew 64% (n = 12) of tumors as single-generation PDXs [20]. In a subsequent study of high-risk prostate cancer, including familial prostate cancer, the take rate for single-generation PDXs was 48% (n = 37) [24]. Using these PDXs, we studied the response to short-term (3 days-4 weeks) castration, and identified residual populations of quiescent tumor cells. These cells had regenerative potential because they regrew as rapidly proliferating tumors after testosterone was readministered. Zhao and colleagues also detected castration-tolerant cancer cells in first-generation grafts of primary prostate adenocarcinoma, including high-risk primary prostate cancer, following 1 to 2 months of castration, with tumors restored after androgen repletion [25, 26]. Thus, the one-time PDXs are valuable research tools for identifying cellular subpopulations and studying tumor growth in a manipulable in vivo model, providing insights that are difficult to obtain from patient specimens or cell culture studies.

## 5. Knowing What’s Growing in Your Patient-Derived Xenograft

Whether establishing a new collection of one-time or serially transplantable PDXs or maintaining existing models, it is essential to rigorously adopt the rule to “*know what you grow.*” Similar to previous experience with cell lines, where there are well known examples of misclassification [27], incorrect descriptions of PDXs can mislead the interpretation of results. The excitement of successfully establishing a new PDX quickly dissipates if it does not represent the intended tumor type. This can occur when human lymphoma arises within grafts, as has been reported for PDXs of a wide variety of tumors, including breast and prostate cancer [19, 28-32]. These lymphomas likely develop from lymphocytes that are present in the original patient tissues and can proliferate in the permissive environment of immunocompromised host mice [33]. Most lymphomas that occur in PDXs are Epstein-Barr virus–positive β-cell lymphomas, although a few T-cell lymphomas have been reported [19, 30]. The telltale signs of lymphoma include splenomegaly of the host mice, rapid and metastatic growth of grafts, lymphoid pathology, and detection of CD45, CD20, and Epstein-Barr virus within grafts [19, 28-31, 33]. In addition to human lymphoma, spontaneous murine malignancies occasionally arise in host mice, and may be confused with PDXs [34, 35]. Both cases demonstrate that it is necessary to regularly review the pathology of PDXs to ensure they are not misclassified.

Even PDXs with the correct pathology can be misidentified. In large collections of PDXs, where tumors are continually being regrafted, there is an ongoing risk that different tumors may be switched or cross-contaminated. For example, cross-contamination was identified in a cohort of PDXs of pediatric acute lymphoblastic leukemia after several transplantations [36]. Like cell lines, PDXs can be authenticated by comparing their profiles of single-nucleotide variations or short tandem repeats to reference samples, such as germline DNA, tumor tissue, or an earlier-generation PDX [36].

Once a PDX is established and validated, it is also important to thoroughly characterize its histopathology. This provides a deeper understanding of its features and, potentially, the underlying genomic alterations that are present. For PDXs of hormone-dependent cancers, a useful panel for immunohistochemistry would include markers of epithelial cell types (eg, cytokeratin 8/18, high-molecular-weight cytokeratin, p63, smooth muscle actin), neuroendocrine markers (chromogranin A, synaptophysin, neuron-specific enolase, CD56), common tumor markers (AMACR, prostate-specific antigen, prostate‐specific membrane antigen, ERG), and steroid hormone receptors (AR, ERα, progesterone receptor, and splice variants) [17, 21, 37, 38]. Beyond staining with these markers, it is prudent to ensure that a pathologist examines the growth pattern of tumor cells within PDXs [6]. For example, some AR-positive prostate adenocarcinomas can have ductal rather than acinar morphology, and this pathology can signify a different profile of genomic alterations [39]. Altogether, the histopathological features of a PDX help pinpoint the specific type of patient tumors it represents. They can also reveal surprising new information about the original patient tumor. In our hands, detailed pathology review of PDXs, even first-generation grafts, has uncovered pathologies that were overlooked at the time of patient diagnosis, such as rare cases of de novo neuroendocrine prostate cancer [21].

## 6. Integrating Patient-Derived Xenografts With Other Patient-Derived Models

There is no perfect patient-derived model, so in preclinical research it is useful to integrate different models, each with particular strengths and weakness. In addition to PDXs, patient tumors can be grown as explants, also known as slice cultures, which are whole pieces of tissue cultured ex vivo on sponges or filters [40-42]. Patient tumors can also be grown as organoids, digested cells cultured in vitro in suspension or in matrices, such as hydrogels and Matrigel (Corning Life Sciences) [43-45]. Organoids of cancer cells are also sometimes referred to as *spheroids*, *tumoroids*, or *canceroids* to recognize that they typically contain only one cell type. In vitro cultures can also be established from patient tissues using conditional reprogramming, where epithelium is grown on irradiated mouse fibroblasts and treated with a rho kinase inhibitor [46].

As renewable sources of human tissue, serially transplantable PDXs can be used as starting material for explants, organoids, conditionally reprogrammed cells, and co-cultures with other cell types [21, 47-54] (see Fig. 1). PDXs tissues are useful for optimizing these protocols because they provide a consistent and abundant supply of the same tumors. Indeed, PDX tissue was used to identify culture conditions and growth media for maximizing the proliferation of prostate cancer cells in explants [48, 55]. Similarly, digested PDX tissues were used to compare the growth of prostate cancer organoids in numerous different formulations of media, showing that modified media improved the growth of some tumors [47].

PDXs are also convenient sources of tissue when appropriate patient samples are not otherwise available for testing a specific hypothesis. Just as it is essential to distinguish between different subtypes of breast cancer when designing experiments, it is important not to conflate castrate-sensitive and castrate-resistant prostate cancer. Yet, a shortage of models can lead researchers to use those that are available, but not necessarily the most suitable. PDXs can help overcome this challenge. For example, in prostate cancer research explants are usually limited to primary patient tumors because it is more challenging to obtain sufficient tissue from metastases, whereas organoids are limited to cells from metastases because cultures of primary tumors often become contaminated with benign epithelium [42, 56-58]. In both cases, PDXs may provide a way to select the most suitable tumor for a study, one that represents the desired pathology, genomic features, and stage of disease progression [21, 47, 48].

The integration of different patient-derived models also expands the scope of experimental design for testing a specific hypothesis. Compared to 2D cultures of cell lines, PDXs are low throughput, but explants and organoids bridge this gap by providing way to increase the scale of experiments [59]. For example, organoids from PDXs of prostate cancer have been used to screen a large library of therapeutics, showing that HSP90 inhibitors consistently inhibited the growth of multiple tumors [60]. Explants and organoids are also useful models for short-term experiments, such as examining rapid changes in cell signaling after drug treatment [21]. Therefore, PDXs, explants, and organoids are complementary models, helping researchers to use the right experiments and the right tumors to investigate scientific questions.

## 7. Patient-Derived Xenografts as Models of Tumor Heterogeneity

All PDXs, including one-time grafts, slow-growing PDXs that do not necessarily meet the criteria for drug screening, and rapidly growing PDXs that are distributed by consortia, can be interrogated in depth to provide an enormous amount of data about intertumor and intratumor heterogeneity.

Sophisticated molecular studies have been conducted with breast cancer PDXs [16, 61, 62], and similar reports are emerging from laboratories with larger collections of prostate cancer PDX. For example, the LuCAP series of 21 PDXs have genomic and phenotypic features representing human disease, including amplification of the AR, *PTEN* deletion, *TP53* deletion and mutation, *RB1* loss, *TMPRSS2-ERG* rearrangements, *SPOP* mutation, hypermutation due to *MSH2/MSH6* genomic aberrations, and *BRCA2* loss [63, 64]. The MD Anderson prostate cancer PDX series (MDA PCa PDX) also captures the molecular landscape of *AR* alterations, *ERG* fusions, and *PTEN* loss, as well as rare or unappreciated mutations, such as a focal deletion of the *SPOPL* gene, providing unique models to study the significance of specific genomic alterations in prostate tumors [65]. Similar molecular data are available for the Living Tumor Laboratory series of prostate cancer PDXs, with profiling of long noncoding RNAs [66, 67], microRNAs [68], and genes involved in cellular energetics and macromolecular biosynthesis [69], providing an expansive transcriptomic data set from these informative models. This application of PDXs for discovery research, beyond preclinical testing, offers a powerful resource for studying unique oncogenic drivers, mechanisms of de novo and acquired drug resistance, novel biomarkers, and new therapeutic targets.

Because the purpose of PDXs is to model human disease, many studies have examined their fidelity to the original patient specimens. Overall, PDXs largely maintain the pathology, molecular features, and therapeutic sensitivity of patient tumors [14, 61, 70-72]. Yet, deep genomic profiling has detected clonal selection and new alterations in PDXs, including of breast cancer [73, 74]. Similar observations have been reported for other patient-derived models [75]. Differences between PDXs and patient samples might arise through intratumoral heterogeneity of the original tissue, genomic instability in rapidly proliferating tumor cells, and the selective pressure of establishing and regrafting tumors in immunocompromised mice [75]; however, no systematic, PDX-specific genomic alterations were identified in a recent study by the PDXNET and EurOPDX consortia [61, 72]. Once again, the maxim “know what you grow” applies. PDXs need to be characterized carefully, not just when they are established, but as they are used over time.

Tumor heterogeneity in PDXs may be a challenge if they are to be used for personalized medicine, but it can also be an opportunity. By establishing different PDXs from the same tumor or the same patient, it is possible to reveal the preexisting heterogeneity in patient samples and investigate how variations in molecular features affect tumor phenotypes [21, 32, 63, 65]. For example, the Living Tumor Laboratory established 5 PDXs with varying metastatic potential from a single primary tumor of high-grade prostate cancer [76]. Subsequent genomic and transcriptomic analyses showed that their phenotype was associated with a distinct gene expression signature in the mouse stroma [76]. Patient-matched PDXs can also be established from an individual patient at different stages of disease progression [61, 77] or from different metastases [21, 78, 79]. Indeed, several sets of patient-matched PDXs of prostate cancer and other malignancies have been established with tissues from rapid autopsy programs, which often provide multiple metastases from each patient [21, 78-81].

Tumor heterogeneity can also be experimentally induced among PDXs through the selective pressure of treatment. PDXs of prostate cancer are often treated by castrating host mice. This mimics the reduction in circulating androgen levels in patients being treated with the AR pathway inhibitor abiraterone acetate [82]. Sublines have been established from PDXs of prostate cancer by maintaining them in testosterone-supplemented versus castrated host mice, providing useful models to study changes in AR signaling in castration-resistant prostate cancer [32, 63, 83, 84]. Similarly, different sublines of luminal breast cancer PDXs have been established through ovariectomy and taxoxifen treatment of host mice [85]. Therefore, purposely inducing changes in PDXs can generate new models for studying tumor biology.

## 8. Summary

Patient tumors are valuable resources for research discovery and drug testing, and growing them as PDXs provides the tools for generating new knowledge. Yet, PDXs come in different guises; they can be used one-time as first-generation grafts, or serially transplanted to be used and shared between laboratories for many years.

For one-time as well as serially transplantable PDXs, and for whatever purpose they are used, the fundamental rule is to know what you grow. This is no different from ensuring the accuracy of other laboratory techniques, and is critical for interpreting results. Pathology, authentication, and fidelity are all aspects of accurately characterizing PDXs. If, for example, a PDX has a mixed pathology, each pathology may have a different molecular profile, and one might respond to treatment, whereas the other may be resistant. Authentication verifies that PDXs are not misclassified or cross-contaminated, avoiding misinterpretation of results. Fidelity, the degree of similarity between a PDX and the original tumor, may be essential if a particular PDX is to be used to inform a patient’s treatment, assuming it can be established and treated in a suitable time frame. However, in many cases, patients who donate breast or prostate cancer tissue to PDX programs undergo disease progression before the PDXs are successfully established. In this case, as long as PDXs are well characterized, their fidelity is less imperative for discovery research, for which PDXs represent subgroups of patients, rather than specific individuals. Furthermore, by knowing what is growing, any deviations from the original patient specimens may be informative about mechanisms of resistance.

Preclinical models of hormone-dependent tumors, such as breast and prostate, are essential tools of the trade for research. PDXs are one such tool, and they complement other models. Altogether, PDXs, cell lines, explants, and organoids are all needed in a researcher’s toolbox. The ability to share serially transplantable PDXs is critical to their use in global consortia, but it is worth remembering that one-time PDXs can also yield invaluable information, especially for tumors that are difficult to grow, such as prostate cancer. The final question we pose is, if we generate PDXs and then share them, how do we ensure that the recipient laboratories have the expertise, training, and infrastructure to use them? These challenges can force laboratories to fall back on using cell lines, which do not represent the complexity of patient tumors. Increasing the use of PDXs will require generosity from the leaders in the field and adaptability by laboratories that apply these contemporary approaches to address their research questions.

## Data Availability

Data sharing is not applicable to this article because no data sets were generated or analyzed during the present study.

## Abbreviations

AR: androgen receptor
ER+: estrogen receptor positive
JAX: the Jackson laboratory
kConFab: Kathleen Cuningham Foundation Consortium for Research into Familial Aspects of Breast Cancer
PCT: PDX clinical trials
PDX: patient-derived xenografts
TNBC: triple-negative breast cancer

## Additional Information

***Disclosure Summary:*** The authors have nothing to disclose.
